# Salivary Secretory Immunoglobulin a secretion increases after 4-weeks ingestion of chlorella-derived multicomponent supplement in humans: a randomized cross over study

**DOI:** 10.1186/1475-2891-10-91

**Published:** 2011-09-09

**Authors:** Takeshi Otsuki, Kazuhiro Shimizu, Motoyuki Iemitsu, Ichiro Kono

**Affiliations:** 1 Faculty of Health and Sport Sciences, Ryutsu Keizai University, Ryugasaki, Ibaraki, Japan; 2 Sports Research & Development Core, University of Tsukuba, Tsukuba, Ibaraki, Japan; 3 Faculty of sport and health science, Ritsumeikan University, Kusatsu, Shiga, Japan; 4 University of Tsukuba, Tsukuba, Ibaraki, Japan

## Abstract

**Background:**

Chlorella, a unicellular green alga that grows in fresh water, contains high levels of proteins, vitamins, minerals, and dietary fibers. Some studies have reported favorable immune function-related effects on biological secretions such as blood and breast milk in humans who have ingested a chlorella-derived multicomponent supplement. However, the effects of chlorella-derived supplement on mucosal immune functions remain unclear. The purpose of this study was to investigate whether chlorella ingestion increases the salivary secretory immunoglobulin A (SIgA) secretion in humans using a blind, randomized, crossover study design.

**Methods:**

Fifteen men took 30 placebo and 30 chlorella tablets per day for 4 weeks separated by a 12-week washout period. Before and after each trial, saliva samples were collected from a sterile cotton ball that was chewed after overnight fasting. Salivary SIgA concentrations were measured using ELISA.

**Results:**

Compliance rates for placebo and chlorella ingestions were 97.0 ± 1.0% and 95.3 ± 1.6%, respectively. No difference was observed in salivary SIgA concentrations before and after placebo ingestion (*P *= 0.38). However, salivary SIgA concentrations were significantly elevated after chlorella ingestion compared to baseline (*P *< 0.01). No trial × period interaction was identified for the saliva flow rates. Although the SIgA secretion rate was not affected by placebo ingestion (*P *= 0.36), it significantly increased after 4-week chlorella ingestion than before intake (*P *< 0.01).

**Conclusions:**

These results suggest 4-week ingestion of a chlorella-derived multicomponent supplement increases salivary SIgA secretion and possibly improves mucosal immune function in humans.

## Introduction

Protein-calorie [1-3], vitamin [4-6], iron [7], and folate [8] malnutrition has been reported to cause immune deficiencies. Undernutrition is not unique to developing nations. Furthermore, in developed countries, the total food energy intake decreases with age [9] because of social [10], psychological [11,12], and medical factors [13]. The nutritional status of young adults declines because of various factors such as skipping breakfast [14,15], relying on fast food [16,17], and dieting to achieve a thin body [18,19]. One of the most efficient methods to improve the nutritional status is use of dietary supplements. However, it is not easy for the general public to choose suitable supplements for improving their individual nutritional status. For individuals with inadequate eating habits, multicomponent dietary supplements that can improve the overall immune function may be beneficial.

Chlorella, a unicellular green alga that grows in fresh water, contains high levels of proteins, chlorophylls, vitamins, minerals, and dietary fibers. This is an important advantage as an ingredient of dietary supplement. It is possible that the multicomponent dietary supplement acts to correct some underlying nutrient/nutrients deficiency and hence gets the positive outcomes such as immunoenhancing effects. Indeed, chlorella-derived supplements are widely used [20] and believed to enhance immune function [21,22]. A previous study has investigated serum antibody titers against influenza and shown the immunoenhancing effects of chlorella ingestion in middle-aged healthy adults [22]. Furthermore, supplementation with chlorella has been reported to increase the concentration of secretory immunoglobulin A (SIgA) in breast milk [21]. However, to the best of our knowledge, no studies regarding the effects of chlorella supplementation on salivary or intestinal-fluid SIgA have been reported. SIgA plays a crucial role in mucosal immune function and is the first line of defense for the human body against pathogenic microbial invasion [23]. Previous studies have reported an association between SIgA concentrations and risks of infection [24-26]. Therefore, it is of interest whether oral supplementation with chlorella can increase the salivary or intestinal-fluid SIgA secretion.

We hypothesized that ingestion of a chlorella-derived multicomponent supplement enhances mucosal immune functions. To test this hypothesis, we investigated the effects of 4-week placebo/chlorella supplementation on salivary SIgA secretion in young men using a blind, randomized, crossover study design. In the previous studies regarding the immunoenhancing effects of chlorella-derived dietary supplements, the placebo capsule was made from microcrystalline cellulose [22] or no restriction was imposed on the control group [21]. According to these studies, we attempted to make a large difference in nutritive values between the placebo and chlorella tablets based on the idea that the advantage of chlorella as a dietary supplement is to contain various nutrients.

## Methods

### Participants

Fifteen men volunteered to participate in this study; none of them used dietary supplements on a regular basis. The participants were asked not to change their regular lifestyles while taking the placebo/chlorella tablets during the experimental periods. None of the participants had any sign, symptom, and history of overt chronic diseases. Furthermore, none of them was taking any medication and had a history of smoking. The mean (± SE) age and height values were 20.4 (0.3) y and 1.68 (0.01) m, respectively.

The present study was approved by the Ethical Committee of the Institute of Health and Sport Sciences of the University of Tsukuba. This study conformed to the principles outlined in the Helsinki Declaration. All participants gave their written informed consent before inclusion in this study.

### Experimental design

The participants took part in two trials -placebo and chlorella- in a randomized order. Saliva samples were obtained from the participants after overnight fasting according to methods described in our previous studies [27,28]. From next day, the participants took 30 placebo or 30 chlorella (SunChlorella A; SunChlorella, Kyoto, Japan) tablets per day for 4 weeks. Saliva samples were collected again on the day following the final ingestion. Compliances with the prescription were documented via ingestion logs. After a washout period of at least 12 weeks, the second trial commenced and the procedures were same as those in the first trial.

### Placebo and chlorella tablets

The mass of the placebo tablet was 243 mg/tablet and that of the chlorella tablet was 200 mg/tablet. The placebo tablets were made according to following procedures. First, a blend of lactose (82.5%) and colorant (17.5%) was dissolved in purified water and dried. Next, this dry matter (95%) was mixed with sucrose fatty acid ester (5%) and converted to tablets. The chlorella tablets contained dried chlorella pyrenoidosa powder as the main ingredient. Nutritional values of these tablets are shown in Table 1.

**Table 1:** Nutritional values of placebo and chlorella tablets.

	Placebo	Chlorella
Energy, kcal/100 g	406	399
Moisture, g/100 g	3.2	5.3
Protein, g/100 g	2.0	60.8
Lipid, g/100 g	5.9	9.2
Saccharide, g/100 g	85.6	6.3
Dietary fiber, g/100 g	1.1	11.9
Ash, g/100 g	2.2	6.5

### Saliva samples

Before the samplings, subjects refrained from alcohol consumption and intense physical activity (exercise) for 24 h to avoid immediate (acute) physiological effects. After overnight fasting, saliva samples were obtained in a quiet, temperature-controlled room as previously described [27,28]. Briefly, participants rinsed their mouths with distilled water (30 s × 3 times) and then rested for at least 5 min. Saliva production was stimulated by chewing sterilized cotton (Salivette; Sarstedt, Nümbrecht, Germany) at a frequency of 60 chews/60 s. The amount of saliva in grams was converted to milliliters assuming a saliva density of 1 g/mL. The obtained saliva samples were separated from the cottons by centrifuging at 1,460 *g*. After measurement of the sample volume, saliva samples were frozen at -60°C. We measured salivary SIgA concentrations using ELISA in accordance with the procedures reported in our previous studies [27,28]. The SIgA secretion rate (*μ*g/min) was calculated by multiplying the absolute SIgA concentration (*μ*g/mL) with the saliva flow rate (mL/min).

### Statistical analysis

Data are expressed as mean ± SE. Differences in the actual periods of supplementation and compliances with the ingestion protocol between the placebo and chlorella trials were tested using an unpaired *t-*test. To compare the effects of ingestion between the placebo and chlorella trials, statistical analysis was performed using repeated-measures two-way ANOVA (trial × period). In the case of a significant trial × period interaction, a post hoc test (Bonferroni-Dunn) was used to identify the effect of placebo/chlorella ingestion. *P *< 0.05 was accepted as significant.

## Results

The periods of ingestion did not differ between the placebo (4.2 ± 0.1 wk) and chlorella (4.2 ± 0.2 wk) trials (*t *= 0.3, *P *= 0.73). Furthermore, compliances were comparable between the placebo (97.0 ± 1.0%) and chlorella (95.3 ± 1.6%) trials (*t *= 0.9, *P *= 0.36). Table 2 shows the body weights, lean body weights, body fat percentages, and body mass indices of participants before and after oral supplementation. No trial × period interaction was observed in these indices (body weight, *F *= 0.2 and *P *= 0.69; lean body weight, *F *= 0.2 and *P *= 0.67; body fat percentage, *F *= 0.0 and *P *= 0.98; body mass index, *F *= 0.1 and *P *= 0.70).

**Table 2:** Body weight, lean body weight, body fat percentage, and body mass index before and after oral supplementations with placebo/chlorella.

		Body weight, kg	Lean Body Weight, kg	Body fat, %	Body Mass Index
Placebo	Before	65.1 ± 2.1	52.9 ± 1.5	18.3 ± 1.6	23.1 ± 0.8
	
	After	65.7 ± 2.2	53.3 ± 1.4	18.3 ± 1.7	23.3 ± 0.7

Chlorella	Before	64.7 ± 2.3	52.7 ± 1.4	17.9 ± 1.7	22.9 ± 0.8
	
	After	65.1 ± 2.3	53.0 ± 1.4	17.9 ± 1.7	23.0 ± 0.8

Values are means ± SEM.

**Figure 1:**
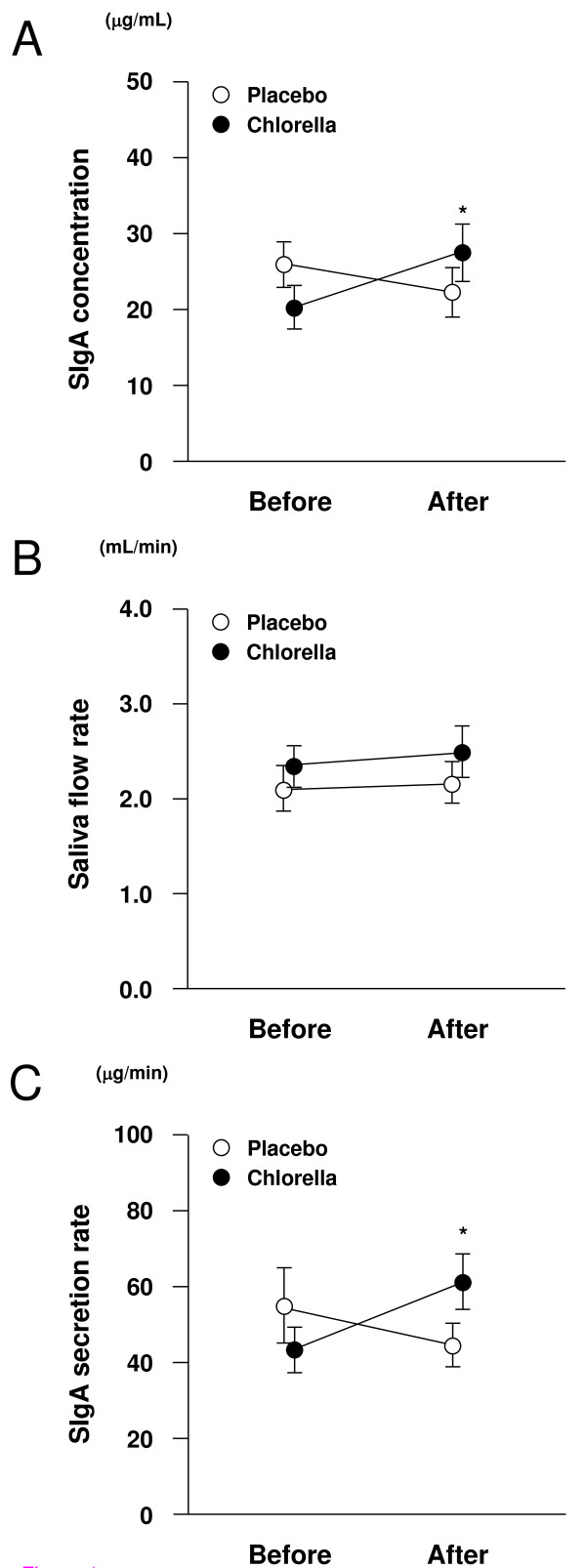
**Effects of supplementation with chlorella-derived multicomponent supplement on mucosal immune function**. These figures show salivary secretory immunoglobulin A (SIgA) concentrations (A), saliva flow rates (B), and salivary SIgA secretion rates (C) before and after 4-week placebo (*n *= 15)/chlorella (*n *= 15) ingestion. While no difference was observed in salivary SIgA concentrations and secretion rates between before and after placebo supplementation, these indices significantly increased after chlorella intake compared to baseline. The saliva flow rates did not change after supplementation with placebo or chlorella compared to those before intervention. Data are expressed as mean ± SE. *, significant change compared to before ingestion.

Figure 1A demonstrates salivary SIgA concentrations before and after 4-week placebo/chlorella supplementation. Trial × period interaction was identified for salivary SIgA concentrations (*F *= 5.9, *P *= 0.02). Oral supplementation with placebo did not affect salivary SIgA concentration (*P *= 0.38), but its concentration significantly increased after chlorella ingestion compared to that before ingestion (*P *< 0.01).

As described in Figure 1B, no trial × period interaction was identified for saliva flow rates (*F *= 0.2, *P *= 0.64).

SIgA secretion rates before and after 4-week placebo/chlorella ingestion are shown in Figure 1C. ANOVA revealed a trial × period interaction (*F *= 5.3, *P *= 0.02). While no difference was observed in salivary SIgA secretion rates between before and after placebo supplementation (*P *= 0.36), it significantly increased after chlorella intake compared to baseline in multiple comparisons (*P *< 0.01).

## Discussion

We investigated the effects of 4-week ingestion of a chlorella-derived multicomponent supplement on salivary SIgA secretion using a blind, randomized, crossover study design. This is a first study to evaluate the effects of chlorella-derived supplement on mucosal immune functions in humans. The salient finding of this study is that both salivary SIgA concentration and secretion rate increased after 4-week chlorella supplementation compared to baseline. Similar changes were not identified in the placebo trial. We therefore concluded that oral supplementation with a chlorella-derived multicomponent supplement enhances mucosal immune function.

Salivary SIgA secretion was used to investigate the immunoenhancing effect of ingestion of a chlorella-derived multicomponent supplement. Salivary SIgA is the first line of defense against respiratory tract infections such as pneumonia and influenza [23]. Klentrou *et al*. [24] reported that an increase in salivary SIgA concentration following 12 weeks of moderate exercise was related to a decrease in a number of sick days. Furthermore, Gleeson *et al*. [25] reported that a mean salivary SIgA concentration during a 7-month training period of elite swimmers was associated with a number of infections contracted during this period. Therefore, we believe that salivary SIgA concentrations and secretion rates are valid indices of mucosal immune function.

Several factors are responsible for reductions in nutrients and calorie intakes. Many studies have pointed out that a diminished sense of smell and taste, increased cytokine activity, altered gastrointestinal function, and altered hormone secretion may induce anorexia even in healthy older adults [29,30]. Further, adverse social factors such as loneliness [10], psychological factors such as depression [11,12], and medical factors such as poor dentition [13] may also cause undernutrition in older adults. In young adults, contributing factors include skipping breakfast [14,15], relying on fast food [16,17], and dieting to achieve a thin body [18,19]. In particular, university students living away from home and those who have a sedentary lifestyle are at a higher risk of poor nutrition than students who live at home and participate in sporting activities [31]. The majority of the participants in this study were university students. There exists a possibility that the participants had inadequate nutritional status and the nutrients in the chlorella-derived supplement attenuated their health problems. We can speculate, from lean body weight, body fat, and body mass index, that total calorie and protein intakes in the subjects were not insufficient. However, we could not investigate their eating habits and perform any blood chemical analysis. One of the next steps is to clarify the mechanisms responsible for the chlorella ingestion-induced increase in salivary SIgA secretion.

This study has following limitations. First, we could not investigate eating habits and perform any blood chemical analysis as mentioned above. Second, we have no data describing clinical significance of change in salivary SIgA secretion. Although it is possible that the dietary supplement-related additional antibodies in healthy young humans elevate a reserve of immune function, it may not improve their infection rate at normal condition. Intervention studies in humans with reduced salivary SIgA level like athletes during training camp and older humans are needed. This study is an initial step to elucidate the effects of chlorella-derived supplement on mucosal immune functions. Recently, Yamauchi et al. [26] reported an expression of Epstein-Barr virus-DNA in saliva and an increase in a number of upper respiratory symptoms occurred on the following day of approximately 23 percent reduction in salivary SIgA secretion rate, although this reduction was smaller than the increase by the chlorella supplementation in our study (41%). Again, the salivary SIgA secretion rate after the chlorella ingestion was 37 percent greater than that after the placebo intake. Therefore, we consider the elevation of salivary SIgA secretion rate in the chlorella trial was clinically significant. Third, we obtained saliva in the morning to equalize the conditions of subject among four sampling points (placebo or chlorella trials × before and after ingestions) although salivary SIgA level is not stable in the morning especially during 10 min after awakening [32]. The saliva collection in this study was performed at least 1 hour after awakening, however, we can not rule out the effect of diurnal cycle in salivary SIgA secretion.

## Conclusion

We concluded that ingestion of a chlorella-derived multicomponent supplement increases salivary SIgA secretion and possibly enhances mucosal immune function.

## Competing interests

SunChlorella Co., Ltd. provided funding for the study and supplied the test supplements used in the study. TO has received speaker's honorarium from SunChlorella Co., Ltd. KS, MI and IK have no competing interests.

## Authors' contributions

TO and KS designed the research. TO, KS and MI conducted the research. TO performed the statistical analysis and wrote this paper with support from KS, MI and IK. All authors read and approved the final manuscript.

## References

[B1] McMurray DN Rey H Casazza LJ Watson RR Effect of moderate malnutrition on concentrations of immunoglobulins and enzymes in tears and saliva of young Colombian children Am J Clin Nutr 1977 30 1944 1948 930866 10.1093/ajcn/30.12.1944

[B2] Watson RR McMurray DN Martin P Reyes MA Effect of age, malnutrition and renutrition on free secretory component and IgA in secretions Am J Clin Nutr 1985 42 281 288 3927700 10.1093/ajcn/42.2.281

[B3] Sirisinha S Suskind R Edelman R Asvapaka C Olson RE Secretory and serum IgA in children with protein-calorie malnutrition Pediatrics 1975 55 166 170 804157

[B4] Gangopadhyay NN Moldoveanu Z Stephensen CB Vitamin A deficiency has different effects on immunoglobulin A production and transport during influenza A infection in BALB/c mice J Nutr 1996 126 2960 2967 9001362 10.1093/jn/126.12.2960

[B5] Sirisinha S Darip MD Moongkarndi P Ongsakul M Lamb AJ Impaired local immune response in vitamin A-deficient rats Clin Exp Immunol 1980 40 127 135 7389210 PMC1536953

[B6] Hodges RE Bean WB Ohlson MA Bleiler RE Factors affecting human antibody response. V. Combined deficiencies of pantothenic acid and pyridoxine Am J Clin Nutr 1962 11 187 199 13907960 10.1093/ajcn/11.3.187

[B7] Chandra RK Saraya AK Impaired immunocompetence associated with iron deficiency J Pediatr 1975 86 899 902 10.1016/S0022-3476(75)80221-6 1127529

[B8] Gross RL Reid JV Newberne PM Burgess B Marston R Hift W Depressed cell-mediated immunity in megaloblastic anemia due to folic acid deficiency Am J Clin Nutr 1975 28 225 232 1119420 10.1093/ajcn/28.3.225

[B9] Centers for Disease Control and Prevention (CDC) Daily dietary fat and total food-energy intakes-- Third National Health and Nutrition Examination Survey, Phase 1, 1988-91 MMWR Morb Mortal Wkly Rep 1994 43 116 117 123-115 8309459

[B10] Walker D Beauchene RE The relationship of loneliness, social isolation, and physical health to dietary adequacy of independently living elderly J Am Diet Assoc 1991 91 300 304 1997551

[B11] Wilson MM Vaswani S Liu D Morley JE Miller DK Prevalence and causes of undernutrition in medical outpatients Am J Med 1998 104 56 63 10.1016/S0002-9343(97)00279-9 9528720

[B12] Smoliner C Norman K Wagner KH Hartig W Lochs H Pirlich M Malnutrition and depression in the institutionalised elderly Br J Nutr 2009 102 1663 1667 10.1017/S0007114509990900 19622192

[B13] Sahyoun NR Otradovec CL Hartz SC Jacob RA Peters H Russell RM McGandy RB Dietary intakes and biochemical indicators of nutritional status in an elderly, institutionalized population Am J Clin Nutr 1988 47 524 533 3348164 10.1093/ajcn/47.3.524

[B14] Lien L Is breakfast consumption related to mental distress and academic performance in adolescents? Public Health Nutr 2007 10 422 428 17362539 10.1017/S1368980007258550

[B15] Tanaka M Mizuno K Fukuda S Shigihara Y Watanabe Y Relationships between dietary habits and the prevalence of fatigue in medical students Nutrition 2008 24 985 989 10.1016/j.nut.2008.05.003 18562170

[B16] Driskell JA Meckna BR Scales NE Differences exist in the eating habits of university men and women at fast-food restaurants Nutr Res 2006 26 524 530 10.1016/j.nutres.2006.09.003

[B17] Morse KL Driskell JA Observed sex differences in fast-food consumption and nutrition self-assessments and beliefs of college students Nutr Res 2009 29 173 179 19358931 10.1016/j.nutres.2009.02.004

[B18] Sano A Le DS Tran MH Pham HT Kaneda M Murai E Kamiyama H Oota Y Yamamoto S Study on factors of body image in Japanese and Vietnamese adolescents J Nutr Sci Vitaminol (Tokyo) 2008 54 169 175 18490848 10.3177/jnsv.54.169

[B19] Hayashi F Takimoto H Yoshita K Yoshiike N Perceived body size and desire for thinness of young Japanese women: a population-based survey Br J Nutr 2006 96 1154 1162 10.1017/BJN20061921 17181892

[B20] Kay RA Microalgae as food and supplement Crit Rev Food Sci Nutr 1991 30 555 573 10.1080/10408399109527556 1741951

[B21] Nakano S Takekoshi H Nakano M Chlorella (Chlorella pyrenoidosa) supplementation decreases dioxin and increases immunoglobulin a concentrations in breast milk J Med Food 2007 10 134 142 10.1089/jmf.2006.023 17472477

[B22] Halperin SA Smith B Nolan C Shay J Kralovec J Safety and immunoenhancing effect of a Chlorella-derived dietary supplement in healthy adults undergoing influenza vaccination: randomized, double-blind, placebo-controlled trial CMAJ 2003 169 111 117 12874157 PMC164975

[B23] Lamm ME Nedrud JG Kaetzel CS Mazanec MB IgA and mucosal defense APMIS 1995 103 241 246 10.1111/j.1699-0463.1995.tb01101.x 7612253

[B24] Klentrou P Cieslak T MacNeil M Vintinner A Plyley M Effect of moderate exercise on salivary immunoglobulin A and infection risk in humans Eur J Appl Physiol 2002 87 153 158 10.1007/s00421-002-0609-1 12070626

[B25] Gleeson M McDonald WA Pyne DB Cripps AW Francis JL Fricker PA Clancy RL Salivary IgA levels and infection risk in elite swimmers Med Sci Sports Exerc 1999 31 67 73 10.1097/00005768-199901000-00012 9927012

[B26] Yamauchi R Shimizu K Kimura F Takemura M Suzuki K Akama T Kono I Akimoto T Virus activation and immune function during intense training in rugby football players Int J Sports Med 2011 32 393 398 10.1055/s-0031-1271674 21380978

[B27] Akimoto T Nakahori C Aizawa K Kimura F Fukubayashi T Kono I Acupuncture and responses of immunologic and endocrine markers during competition Med Sci Sports Exerc 2003 35 1296 1302 10.1249/01.MSS.0000078934.07213.25 12900681

[B28] Shimizu K Kimura F Akimoto T Akama T Kuno S Kono I Effect of free-living daily physical activity on salivary secretory IgA in elderly Med Sci Sports Exerc 2007 39 593 598 10.1249/mss.0b013e318031306d 17414795

[B29] Chapman IM Nutritional disorders in the elderly Med Clin North Am 2006 90 887 907 10.1016/j.mcna.2006.05.010 16962848

[B30] Morley JE Anorexia of aging: physiologic and pathologic Am J Clin Nutr 1997 66 760 773 9322549 10.1093/ajcn/66.4.760

[B31] Shimbo S Zhang ZW Matsuda-Inoguchi N Higashikawa K Nakatsuka H Watanabe T Ikeda M Effects of life away from home and physical exercise on nutrient intake and blood/serum parameters among girl students in Japan Tohoku J Exp Med 2004 203 275 286 10.1620/tjem.203.275 15297732

[B32] Hucklebridge F Clow A Evans P The relationship between salivary secretory immunoglobulin A and cortisol: neuroendocrine response to awakening and the diurnal cycle Int J Psychophysiol 1998 31 69 76 10.1016/S0167-8760(98)00042-7 9934622

